# Conserved genomic neighborhood is a strong but no perfect indicator for a direct interaction of microbial gene products

**DOI:** 10.1186/s12859-019-3200-z

**Published:** 2020-01-03

**Authors:** Robert Esch, Rainer Merkl

**Affiliations:** 1 Faculty of Mathematics and Computer Science, University of Hagen, D-58084 Hagen, Germany; 2 Institute of Biophysics and Physical Biochemistry, University of Regensburg, D-93040 Regensburg, Germany

**Keywords:** Protein-protein interaction, Complex formation, Sequence similarity network, Genome neighborhood network, Binary classifier

## Abstract

**Background:**

The order of genes in bacterial genomes is not random; for example, the products of genes belonging to an operon work together in the same pathway. The cotranslational assembly of protein complexes is deemed to conserve genomic neighborhoods even stronger than a common function. This is why a conserved genomic neighborhood can be utilized to predict, whether gene products form protein complexes.

**Results:**

We were interested to assess the performance of a neighborhood-based classifier that analyzes a large number of genomes. Thus, we determined for the genes encoding the subunits of 494 experimentally verified hetero-dimers their local genomic context. In order to generate phylogenetically comprehensive genomic neighborhoods, we utilized the tools offered by the Enzyme Function Initiative. For each subunit, a sequence similarity network was generated and the corresponding genome neighborhood network was analyzed to deduce the most frequent gene product. This was predicted as interaction partner, if its abundance exceeded a threshold, which was the frequency giving rise to the maximal Matthews correlation coefficient. For the threshold of 16%, the true positive rate was 45%, the false positive rate 0.06%, and the precision 55%. For approximately 20% of the subunits, the interaction partner was not found in a neighborhood of ± 10 genes.

**Conclusions:**

Our phylogenetically comprehensive analysis confirmed that complex formation is a strong evolutionary factor that conserves genome neighborhoods. On the other hand, for 55% of the cases analyzed here, classification failed. Either, the interaction partner was not present in a ± 10 gene window or was not the most frequent gene product.

## Background

A fundamental organizational unit of microbial genomes is the operon consisting of a cluster of genes that are transcribed into a single mRNA molecule [1, 2], which allows for the quasi-parallel synthesis of the gene products. Commonly, proteins encoded in the same operon work together, e. g., as enzymes catalyzing subsequent steps of a metabolic pathway. Thus, genomic neighborhood is a reliable indicator for the functional association of proteins and an important element for the generation of functional networks offered by databases like STRING [3].

**Fig. 1:**
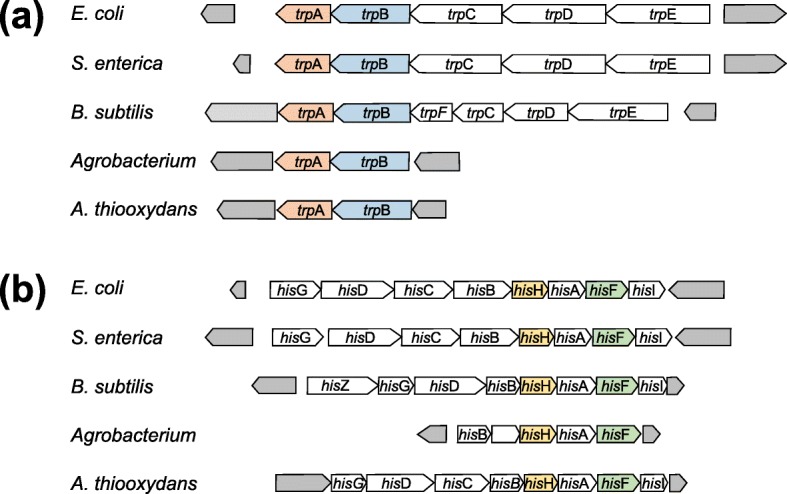
Genomic neighborhood of the *trp*A gene (**a**) and of the *his*F gene (**b**) in five bacterial genomes. The neighborhoods were deduced from the BioCyc database [6] for the genomes of *E. coli* K-12, *S. enterica* enterica A3ES40, *B. subtilis* subtilis 168, *Agrobacterium* sp. H13–3, and *A. thiooxydans* ZJ. The output of BioCyc is shown schematically, but drawn to scale; neighboring genes not related to tryptophan or histidine biosynthesis are filled grey. The gene products of *trp*A and *trp*B and of *his*H and *his*F (all color-coded) form hetero-oligomers, respectively. The conservation of the *trp*A and *trp*B neighborhood in all five genomes suggests the formation of a TrpA/TrpB complex. For the same genomes, the neighborhood of the *his*H gene would more likely propose a HisH/HisA/HisF, a HisH/HisA, or a HisA/HisF, but no HisH/HisF complex. Note that HisH and HisF form a hetero-dimer, whereas HisA is a monomeric protein [7]

An early comparison of nine bacterial and archeal genomes has led to the conclusion that proteins encoded by conserved gene pairs interact physically [4]. An example is the *trp* operon of *Escherichia coli* that consists of the five genes *trp*A – *trp*E catalyzing tryptophan biosynthesis from chorismate [5]. In *E. coli*, *trp*C encodes as a fusion of two genes a bifunctional protein that has TrpC and TrpF functionality. As Fig. 1a shows, the five *trp* genes occur in the same order also in the closely related γ-Proteobacterium *Salmonella enterica* enterica A3ES40 and in the genome of *Bacillus subtilis* subtilis 168, where TrpC and TrpF are encoded by two separate genes. In stark contrast, the BioCyc database [6] indicates that *trp*E lies isolated from other *trp* genes in the genome of *Agrobacterium* sp. H13–3 and that the distance to *trp*A is > 2,200,000 bp. In the genome of *Acidiferrobacter thiooxydans* ZJ, the genomic neighborhood of *trp*E contains *pab*A (a *trp*G homolog), *trp*D, and *trp*C, but the distance to *trp*A is > 50,000 bp. However, in all five genomes, *trp*A and *trp*B are genomic neighbors and the proteins TrpA and TrpB are the two subunits that form the tryptophan synthase, which is a permanent, hetero-oligomeric protein complex that experienced an intricate evolutionary history [8]. These observations propose that a direct protein-protein interaction is an evolutionary factor that preserves genomic neighborhood considerably stronger than a functional interaction of the gene products. Indeed, cotranslational protein assembly and the order, in which the gene products assemble to a complex affect the order of genes in operons [9]; the latter effect is stronger for weakly expressed genes [10].

**Fig. 2:**
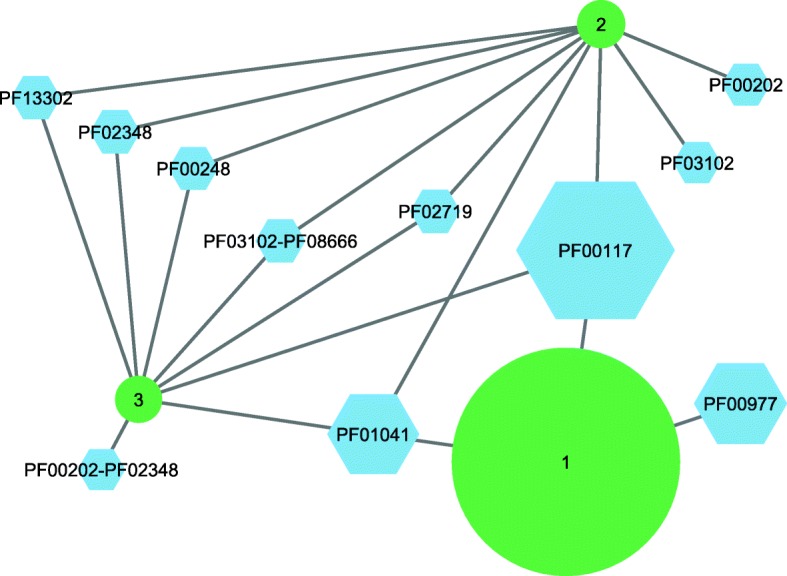
rGNN of InterPro family IPR004651 characterizing the histidine biosynthesis protein HisF. The circles represent three *rep_nodes* 40; their diameter corresponds to the number of corresponding sequences. The hexagons represent *pf_nodes* (i. e., Pfam families) that are labeled with their Pfam-IDs; the size of the hexagons corresponds to the value of *f*(*pf_node*). These *pf_nodes* indicate the most abundant protein functions encoded in the ± 10 genomic neighborhood of the HisF sequences. PF00117 (glutamine amidotransferase I, HisH) was correctly identified as interaction partner of HisF, because it had the largest value ${f}_{\mathrm{max}}^{\ast}\left( pf\_ node\right)$, which was 43.6%. To reduce complexity, all nodes representing ≤ 3 sequences were eliminated for this illustration

On the other hand, the comparison of *his* operons that consist of the genes leading to the synthesis of histidine [11] indicates some intricacies that may complicate a too simplistic inference of direct protein interactions. The *his* operon of *E. coli* contains eight genes and only the *his*H and the *his*F gene products form a hetero-dimer, whereas all other gene products are monomers. As shown in Fig. 1b, the comparison of the *his* operons from the five species introduced above, makes clear that their *his*H and *his*F genes are no immediate neighbors, but are separated by *his*A, which encodes a monomeric enzyme [7]. If this neighborhood of the three genes *his*H, *his*A, and *his*F is conserved in many genomes, it is not possible to deduce in silico the formation of the HisH/HisF complex.

In literature, we did not find a comprehensive characterization of a classifier that predicts the subunits of microbial protein complexes by analyzing genomic neighborhoods. Due to the more than 200,000 sequencing projects listed in the GOLD database [12], we expected a statistically comprehensive sampling of neighborhoods that is sufficient to determine the reliability of such a classifier. We concentrated on the assessment of experimentally verified hetero-dimers based on the analysis of large sets of genomes and addressed two specific questions:

1. How often are microbial proteins that form hetero-dimeric complexes encoded in close genomic vicinity?
2. How reliable is the prediction of hetero-dimeric protein complexes based on neighborhood conservation deduced from a comprehensive number of genomes?

Thus, we determined for the subunits of 494 hetero-dimers the genomic neighborhoods from phylogenetically diverse species and assessed the frequency of the most abundant gene products in a ± 10 gene window. It turned out that 80% of the known interaction partners are encoded in a ± 10 neighborhood. Additionally, we predicted for each subunit the product of the most frequent neighbor as direct interaction partner. Applying a threshold that balances false positive and false negative predictions, 485 of the 1087 known interaction partners were correctly identified by choosing the most abundant gene neighbor.

## Results

### Deducing phylogenetically diverse genomic neighborhoods of bona fide bacterial hetero-dimers

We were interested to find out how reliable the genomic neighborhood indicates for a given subunit (*su*) of a heteromeric complex the interaction partner, if we analyze its neighborhood in genomes from many phylogenetically diverse species. This approach relies on two, mutually reinforcing effects: *i*) Prokaryotic genomes are rather unstable [13, 14], only 5–25% of the genes belonging to operons are shared by at least two distantly related species [15]. Thus, in the framework of this analysis, the abundances of those genes must be low that encode in the neighborhood of a given *su* such proteins that are only functionally related or even functionally unrelated to the *su* under study. *ii*) In contrast, if the propensity for a direct protein-protein interaction affects genomic distances, the specific interaction partners must often be neighbors in a large number of phylogenetically diverse genomes and thus stand out through higher abundances.

The prediction method considered here can only be applied to hetero-oligomers. The simplest form of heteromeric complexes are hetero-dimers consisting of two subunits. Thus, we chose a recently compiled set of bacterial hetero-dimers with known crystal structures that do not possess additional interaction partners like DNA [16]. The corresponding PDB [17] entries were analyzed to deduce pairs of complex-forming subunits ($s{u}_i^1$, $s{u}_i^2$) and the corresponding InterPro [18] and Pfam [19] families. These annotations were indispensable for the subsequent analysis (see below); after the elimination of ambiguous cases, 494 pairs of subunits remained. The corresponding PDB-IDs and detailed results are listed in Additional file 1: Table S1.

To create for all $s{u}_i^{\ast }$ proteins neighborhoods that are phylogenetically most comprehensive, we utilized tools offered by the Enzyme Function Initiative (EFI), which were developed to analyze sequence and function space of protein families [20]. The EFI-Genome Neighborhood Tool (EFI-GNT) computes a genome neighborhood network (GNN) for a given sequence similarity network (SSN). This SSN has to be created beforehand by means of the EFI-Enzyme Similarity Tool (EFI-EST). Thus, for each of the $s{u}_i^{\ast }$ under study, we generated an SSN for the InterPro family it belonged to. If delivered by EFI-EST, we processed *rep_node* 80 networks, otherwise *rep_node* 40 files (for details see Methods).

We chose a ± 10 neighborhood (for justification see Methods) and created for each SSN, i. e. *rep_node* file, a refined genome neighborhood network (rGNN) by utilizing a modified version of AGeNNT [21]. In an rGNN, the neighboring gene products are represented by the Pfam families (*pf_nodes*) they belong to and the *pf_node*-specific *SeqCount* values indicate the number of neighborhoods encoding this protein function. The *SeqCount* values were transferred to relative frequencies *f*(*pf_nodes*); see Formula (1). For each $s{u}_i^{\ast }$, we identified the *pf_node* with the highest frequency ${f}_{\mathrm{max}}^{\ast}\left( pf\_ node\right)$, which was assumed to represent the putative interaction partner of $s{u}_i^{\ast }$, if this frequency exceeded a lower threshold. We analyzed only *pf_nodes* occurring with a minimal frequency of 20%; thus, this analysis of rGNNs considered the large number of neighborhoods represented in the InterPro and Pfam databases, but additionally focused to the most frequent protein functions; see Fig. 2 for an example. Interestingly, the median *SeqCount* value for all $s{u}_i^{\ast }$ elements was 133 contributed by 21.5 phylogenetic phyla (median), which testifies for our analysis to a phylogenetically diverse representation of neighborhoods and genomes.

### Assessing the abundance of neighboring interaction partners

Because some of the interacting proteins are composed of more than one domain, they belong to more than one InterPro family. Thus, our PDB-ID ↔ InterPro ↔ Pfam mapping resulted in 1087 subunits $s{u}_i^{\ast }$, for which we knew the interaction partner involved in complex formation. These were the positive cases (P). Moreover, we assumed that these $s{u}_i^{\ast }$ do not form a complex with any of the other proteins encoded in the genomic neighborhood of $s{u}_i^{\ast }$. These proteins summed up to 596,767 negative cases (N) and we used the P and N cases to compute performance values.

There are hetero-dimeric complexes, where the two subunits are not encoded in close genomic vicinity as exemplified by the *B. subtilis* enzymes PabA and TrpE that form the anthranilate synthase [16]. As indicated by BioCyc [6], *pab*A, which is a multipartner enzyme, is part of the pabBAC-sul-folBK-yazB-yacF-lysS transcription unit that starts in the *B. subtilis* genome at base position 82,831, whereas *trp*E belongs to the *trp* operon that begins at base position 2,377,619. Thus, we presumed that the number of false positive assignments decreases, if we introduce a minimal threshold frequency *f*_*min*_ by testing ${f}_{\mathrm{max}}^{\ast}\left( pf\_ node\right)\ge {f}_{\mathrm{min}}$; see Formula (2). If a *pf_node* reaching *f*_*min*_ represented a known interaction partner, it was a true positive (TP), otherwise it was a false positive (FP). All other *pf_nodes* not reaching this threshold were false negative (FN), if representing an interaction partner and true negative (TN), if representing one of the negative cases.

**Fig. 3:**
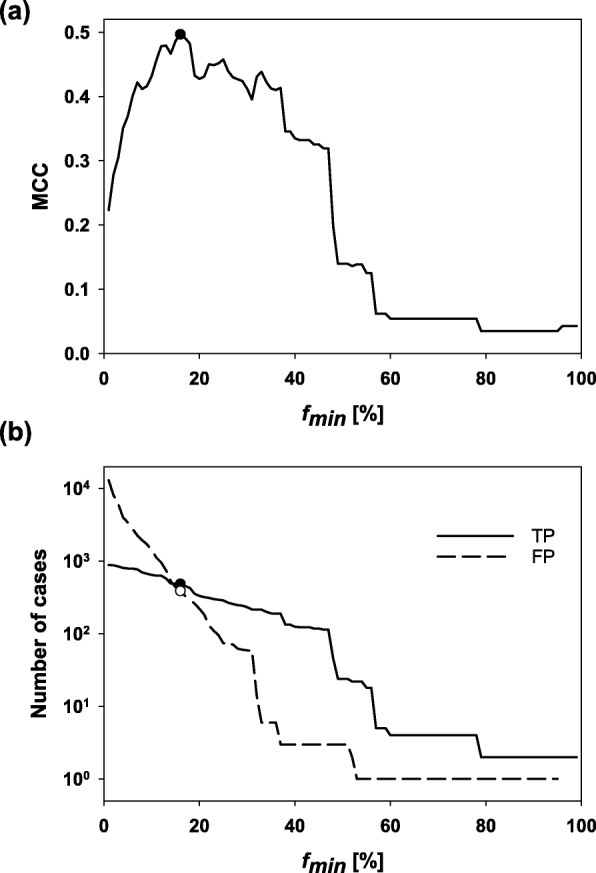
MCC values versus the minimal threshold frequency *f*_*min*_ used to predict interaction partners (**a**). The *f*_*min*_ value (16%) resulting in the highest MCC is indicated by a filled circle. Absolute numbers of TP and FP predictions versus the minimal threshold frequency *f*_*min*_ (**b**). The two circles indicate the number of cases related to the chosen *f*_*min*_ value

The Matthews correlation coefficient (MCC) [22] is considered a fair performance measure even for unbalanced datasets, as it is deduced from all classified cases. Thus, we incremented the threshold *f*_*min*_ in 1% steps between 1 and 100% and determined *f*_*min*_-specific MCC values; see Formula (4). As Fig. 3a indicates, the maximal MCC-value of 0.50 was achieved for *f*_*min*_ = 16%. In this case, 485 of the 1087 known interaction partners were predicted as TP and 389 of the 596,767 putatively non-interacting proteins were FP.

**Fig. 4:**
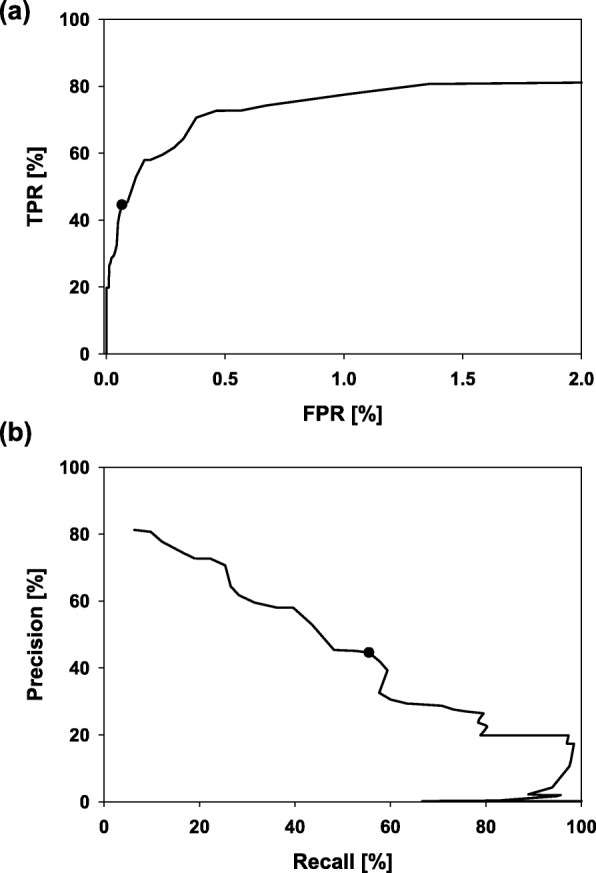
TPR = Recall, FPR, and Precision for *f*_*min*_-values incremented from 0 to 100%. Initial interval of a ROC curve ending at an FPR of 2% (**a**). The Precision/Recall plot for the full range of threshold values (**b**). TPR = Recall (45%), FPR (0.06%), and Precision (55%) values for the classifier operated with *f*_*min*_ = 16% are indicated by filled circles

Figure 3b confirms that the number of FPs is – as expected – negatively correlated with *f*_*min*_, whereas the number of TPs is less affected by the chosen *f*_*min*_ value. Decreasing *f*_*min*_ below 16% would result in more FPs than TPs, which is not desired. Figure 4a represents the initial interval of a TPR versus FPR plot (ROC curve) ending at an FPR of 2%. This rate corresponds to 13,024 FPs, which is far above an acceptable performance. Choosing *f*_*min*_ = 16% results in a TPR of 45% and an FPR of 0.06% (see Formulae (3)); the latter rate must be chosen low due to the enormous number of 596,767 negative cases. Interestingly, the results indicate for this classifier a maximally TPR value of ∼80%. This finding suggests for approximately 20% of the hetero-dimers that the subunits are not encoded in a ± 10 neighborhood. The Precision/Recall curve shown in Fig. 4b indicates a nearly linear anticorrelation between Precision and Recall (i. e., the TPR). For the *f*_*min*_ value suggested by the MCC analysis, Precision is 55% and Recall 45%.

One might argue that smaller neighborhoods reduce the risk of predicting the wrong interaction partner. To address this problem, we have additionally analyzed the ± 3 neighborhood of those 27 cases, where the true interaction partner reached rank two in our prior classification. The results are summarized in Additional file 1: Table S2. For twelve cases (44%), the rank was unaltered; for seven cases (26%), the rank deteriorated below two and for eight cases (30%), the rank increased to one. This finding suggested to us that smaller neighborhoods have no pronounced effect on classification performance.

### If several complexes are encoded in close vicinity, it is difficult to predict interaction partners

Our protocol identified 485 of the 1087 known interaction partners correctly, and we wanted to elucidate reasons for the prediction of 389 FP cases, which are *pf_nodes* (proteins) occurring in the GNNs with a higher frequency than the true interaction partner.

Among the false predictions was the interaction partner of SoxA, which forms together with SoxX the hetero-dimeric SaxAX cytochrome [23]. The gene of SoxY is located in the neighborhood of SaxA at varying positions, but the corresponding *pf_node* had rank three and the neighbors SoxY and SoxZ had higher frequencies, which led to the FP prediction of a SoxA:SoxZ interaction. SoxY and SoxZ form a complex together with SoxB and *sox*VWXYZABCDEFGH is a transcriptional unit in α-Proteobacteria [24]. The STRING database (version 11.0) indicated that the neighborhood of *soxX*, *soxY*, *soxZ*, and *soxA* is conserved in all α- and β-Proteobacteria that possess clear homologs of SoxA. This example illustrates that it is hard to predict the correct interaction partner, if the subunits of more than one complex are encoded in close vicinity.

## Discussion

### Limitations of the current approach

Although the median of the phyla contributing to the *SeqCount* values of the ${f}_{\mathrm{max}}^{\ast}\left( pf\_ node\right)$ nodes was 21.5, one might argue that our approach overestimated the frequencies of functionally related or even functionally unrelated neighbors. The rigorous elimination of genomes from closely related species might reduce this bias, but not the one caused by the horizontal transfer of larger fragments like selfish operons [25]. More efficient would be an elimination method based on the pairwise comparison of the protein functions [26] encoded in the considered neighborhoods. Identical protein functions encoded in highly similar local arrangements would indicate closely related species or cases of horizontal gene transfer. However, this approach would require a rigid preprocessing of the genomes and a completely different software pipeline.

We used a dataset of 1087 subunits to determine the optimal MCC value and identified a threshold *f*_*min*_ of 16% as optimal. If one considers our algorithm as a classifier, one might argue that the algorithm’s parameter were optimized and tested on the same dataset. A cross-validation technique could be used to exclude overfitting. However, as we fixed only one parameter (the *f*_*min*_ value), we consider the risk of overfitting minimal. The dataset analyzed here consists of proteins devoid of non-protein macromolecules that formed complexes with stoichiometries of AB, A_2_B_2_, A_3_B_3_, A_4_B_4_, A_6_B_6_, ABC, and A_2_B_2_C_2_ [16]. As we analyzed only the full set of these proteins, the determined performance values might be misleading for test cases outside our training sample.

## Conclusions

By using EFI services that process large datasets, we have confirmed for hetero-dimers that approximately 45% of the subunits are the most frequent gene products in the GNNs that correspond to a ± 10 neighborhood. Additionally, our data suggest that approximately 20% of the interaction partners are encoded outside of this genomic window.

A survey of the oligomerization state of *E. coli* proteins revealed that hetero-oligomers are a minority: 20% of the proteins are monomers, whereas dimers and tetramers are far more common; 79% of the complexes are homo-oligomers with 2 to 12 subunits and only 21% are hetero-oligomers [27]. Thus, for a comprehensive in silico prediction of all types of protein complexes, a machine learning approach combining several features is required in order to increase classification reliability; for a recent review see e. g. [28].

## Methods

### Mapping PDB entries to InterPro and Pfam families

For the mapping of chains from PDB datasets, the services offered by the European Bioinformatics Institute (EMBL-EBI) were used [29]. The pages “https://www.ebi.ac.uk/pdbe/entry/pdb/*$ID*/analysis” were parsed to determine for the proteins of the PDB dataset with PDB-ID *$ID* the InterPro and Pfam families. The PDB-IDs were taken from a recently prepared dataset [16] consisting of bona fide bacterial hetero-dimers. Mostly due to the co-existence of more than one domain, 186 subunits were mapped to more than one InterPro family, which were all analyzed. Only in one of these cases (chain A of the methylmalonyl-coa mutase PDB-ID 4req) the prediction varied among the assigned InterPro families; compare ranks in Additional file 1: Table S1.

### Creating SSNs and GNNs

Our software pipeline consisted of scripts written in Python [30] that were executed on the compute-server of the EFI or an in-house computer, which were all equipped with Linux. All scripts are deposited at Github.

SSNs were computed command-line based on the EFI cluster for InterPro families with default parameters chosen by EFI-EST. An SSN consists of nodes each representing a sequence; the nodes are interconnected by edges weighted with the BLAST bit score resulting from a pairwise alignment of the related sequences. For large protein families, an extremely high number of edges renders an SSN intractable; thus, EFI-EST does not generate an output file, if the SSN would contain more than 10,000,000 edges. To reduce network complexity, EFI-EST maps sequences sharing at least *x*% sequence identity to one node and generates representative node (*rep_node*) *x* networks.

The SSNs were uploaded to the EFI server for the generation of GNNs. “Raw” GNNs were converted to rGNNs by means of an updated version of AGeNNT [21] that was adapted to the current EFI interfaces. We oriented ourselves on the architecture of the *E. coli* genome and a systematic analysis performed during the design of AGeNNT [21] to determine the window size of the genomic neighborhood to be analyzed. Approximately 80% of all *E. coli* transcription units have fewer than five genes and 80% of all directons, i. e., genes transcribed in the same direction with no intervening one transcribed in the opposite direction, have fewer than ten genes [31]. Moreover, a systematic screening indicated that ± 10 neighborhoods are best suited to identify gene clusters [21]. Thus, we selected a ± 10 neighborhood as default. In this case, the neighborhood consists for each member of the InterPro family of exactly those 20 gene products that are encoded in a ± 10 window. The output of the EFI-GNT is independent of the localization of transcriptional units and represents the function of these proteins by means of PFAM accession numbers.

Consequently, an rGNN consists of *rep_nodes*, i. e., a cluster of sequences from the InterPro family under study and *pf_nodes* representing enzyme functions encoded in the respective neighborhoods. For edges between *rep_nodes* and *pf_nodes*, the coverage is given, which is the relative number of neighborhoods containing the considered enzyme function represented by *pf_ node*. For each *pf_node*, the *SeqCount* parameter indicates the number of genomic neighborhoods possessing this protein function. rGNNs are encoded as xgmml files, which were parsed to deduce from the *SeqCount* values the *pf_node* with the highest frequency ${f}_{\mathrm{max}}^{\ast}\left( pf\_ node\right)$; this one was the candidate for the prediction of interaction partners.

### Classifying *pf_nodes*

For the determination of a relative frequency *f*(*pf_node*), the *pf_node*-specific *SeqCount(pf_node)* value was divided by the sum of all SeqCount(pf_node*) values observed in the rGNN under study, according to

1 $$ f\left( pf\_ node\right)= SeqCount\left( pf\_ node\right)/\sum SeqCount\left( pf\_ node\ast \right) $$

For the assignment of interaction partners, the frequency of the chosen *pf_node* was compared to the threshold *f*_*min*_ according to

2 $$ pred\left( pf\_ node\right)=\Big\{{\displaystyle \begin{array}{l}1\kern1em if\;{f}_{\mathrm{max}}^{\ast}\left( pf\_ node\right)\ge {f}_{\mathrm{min}}\\ {}0\kern1em otherwise\end{array}} $$

and *pf_node* was considered as interacting, if *pred*(*pf_node*) was 1.

### Performance measures

To assess the performance of a classification, we determined the false positive rate (FPR), the true positive rate (TPR = Recall), the Precision

3 $$ FPR=\frac{FP}{N},\kern1em Recall= TPR=\frac{TP}{P},\kern1em Precision=\frac{TP}{TP+ FP} $$

and the MCC value

4 $$ MCC=\frac{TP\times TN- FP\times FN}{\sqrt{\left( TP+ FN\right)\left( TP+ FP\right)\left( TN+ FP\right)\left( TN+ FN\right)}}. $$

In all formulae, P is the number of positive and N the number of negative cases. TP is the number of true positives, TN the number of true negatives, FP the number of false positives, and FN the number of false negatives.

### Visualizing GNNs and rSSNs

All networks were visualized by means of Cytoscape [32].

## Supplementary information

**Additional file 1.** All raw data of the SSN/GNN analyses.

## Data Availability

The code of the software pipeline and the scripts for statistical analysis can be found at https://github.com/merkllab/ConsGNN

## Abbreviations

EFI: enzyme function initiative
EFI-EST: EFI-enzyme similarity tool
EFI-GNT: EFI-genome neighborhood tool
FN: false negative cases
FP: false positive cases
FPR: false positive rate
MCC: Matthews correlation coefficient
N: negative cases
P: positive cases
PDB-ID: protein database identifier
pf_node: a node representing a Pfam family
Pfam-ID: Pfam database identifier
rGNN: refined genome neighborhood network
SeqCount: number of genomic neighborhoods encoding a certain function
SSN: sequence similarity network
su: subunit of a protein complex
TN: true negative cases
TP: true positive cases
TPR: true positive rate
