# Supplementary material for: Conserved genomic neighborhood is a strong but no perfect indicator for a direct interaction of microbial gene products
Source: BMC Bioinformatics. 2020 Jan 3;21:5. doi: 10.1186/s12859-019-3200-z (PMC6941341; doi:10.1186/s12859-019-3200-z)
Supplement: Supplementary file 1 — Additional file 1. All raw data of the SSN/GNN analyses. [file 12859_2019_3200_MOESM1_ESM.pdf]

Conserved genomic neighborhood is a strong but no perfect indicator for a direct interaction of microbial gene products

Robert Esch[2], Rainer Merkl[1*]

[1]Institute of Biophysics and Physical Biochemistry, University of Regensburg,

D-93040 Regensburg, Germany

[2]Faculty of Mathematics and Computer Science, University of Hagen,

D-58084 Hagen, Germany

[*]Corresponding author.

Email addresses

RE: robert.esch@studium.fernuni-hagen.de

RM: rainer.merkl@ur.de

Table S1 Analysis of the full dataset consisting of hetero-dimers. For each PDB-ID of the dataset, all chains were identified and the corresponding InterPro families determined. Additionally, Pfam numbers were deduced for the interaction partner. The column "Sum of Pfam occurrences" gives the sum of the absolute frequencies (*SeqCount* values) of all *pf_nodes* from the GNN generated for the InterPro family listed in column three. The column "Occurrence of Interaction Partner" lists the absolute frequency (*SeqCount* value) of the Pfam node representing the known interaction partner (listed in column four) and the last column lists the normalized frequency of this *pf_node* and its rank. If the known interaction partner was not found in the ±10 neighborhood, the Pfam-ID and the relative frequency of this FP is given. If the GNN contained less than 10 nodes, the number of nodes is additionally listed in the column "Sum of Pfam occurrences". For example, PDB-ID 1AHJ contains eight chains A – H. Chains A, C, E, and G belong to the InterPro families IPR023900 (nitrile hydratase alpha subunit /thiocyanate hydrolase gamma subunit) and IPR018141 (nitrile hydratase, alpha subunit) and the Pfam entry PF02979 (nitrile hydratase, alpha chain). Chains B, D, F, and H belong to InterPro family (nitrile hydratase, beta subunit) and the Pfam entry PF02211 (nitrile hydratase beta subunit). These mappings are listed in the first four columns; note that the Pfam-IDs are given for the corresponding interaction partner. Labeled in red are the cases, where the known interaction partner reached rank 2, resulting in a FN prediction.

**PDB-ID**	**Chains**	**InterPro Family of Chains**	**Pfam of Interaction Partner**	**Sum of Pfam occurrences**	**Occurrence of Interaction Partner**	**Frequency of Interaction Partner [%] (Rank)**
1AHJ	A, C, E, G	IPR023900	PF02211	1508	719	47.7 (1)
	A, C, E, G	IPR018141	PF02211	1339	641	47.9 (1)
	B, D, F, H	IPR003168	PF02979	1390	522	37.6 (1)
1AY7	B	IPR000468	PF00545	3981	788	19.8 (1)
	A	IPR000026	PF01337	2114	1184	56.0 (1)
1B0N	A	IPR010981	PF08671	593	140	23.6 (1)

	B	IPR010981	PF01381	593	140	23.6 (1)
	B	IPR010981	PF08671	593	140	23.6 (1)
1B27, 1B2S, 1B2U, 1B3S, 1BGS, 1BRS,	B	IPR000468	PF00545	3981	788	19.8 (1)
	A	IPR000026	PF01337	2114	1184	56.0 (1)
	A	IPR001887	PF01337	1854	419	22.6 (1)
1DJ7, 2PUO	A	IPR004209	PF02943	1287	0	PF00462 25.0
	A	IPR024707	PF02943	313	0	PF00005 16.0
	B	IPR004207	PF02941	293	0	PF01957 14
1E44, 4UDM	A	IPR003063	PF09000	227	45	19.8 (1)
	B	IPR009105	PF03513	574	74	12.9 (1)
1EAY, 1A0O	A, B	IPR001789	PF09078	-		
	C, D	IPR015162	PF00072	6178	563	9.1 (6)
1EFP	A,C	IPR001308	PF01012	3625	1223	33.7 (1)
	B,D	IPR012255	PF00766	3430	1273	37.1 (1)
	B,D	IPR012255	PF01012	3430	1273	37.1 (1)
1EM8	A,C	IPR007459	-			
	B,D	IPR018382	PF04364	500	0	PF05175-PF08468 7.2
	B,D	IPR004615	PF04364	3887	2	0.05 (>10)
1EWY	A,B	IPR035442	PF00111	449 (9 nodes)	0	PF00485 18.0
	A,B	IPR015701	PF00111	2144	0	PF00378 10.0
	C	IPR010241	PF00175	1238	0	PF11805 5.0
1FFG, 1FFS, 1FFW	A, C	IPR001789	PF09078	-		
	B, D	IPR015162	PF00072	6178	563	9.1 (6)
1KA9	H	IPR010139	PF00977	2277	856	37.6 (1)
	F	IPR006062	PF00117	6166	984	16.0 (1)
	F	IPR004651	PF00117	645	281	43.6 (1)
1U0S	A	IPR010808	PF00072	16625	1118	6.7 (3)
	Y	IPR001789	PF07194			
1V74	A	IPR024440	PF09204	217	95	43.8 (1)
	B	IPR015287	PF11429	707	20	2.8 (1)
1WX2, 1WX4, 1WXC,	A	IPR002227	PF06236	6365	136	2.1 (3)
	B	IPR010928	PF00264	2750	334	12.1 (1)
1WX5	A, C	IPR002227	PF06236	6365	136	2.1 (3)
	B, D	IPR010928	PF00264	2750	334	12.1 (1)
1X1U, 1X1W, 1X1X, 1X1Y 2ZA4	A, B, C	IPR000026	PF01337	2114	1184	56.0 (1)
	A, B, C	IPR001887	PF01337	1854	419	22.6 (1)

	D, E, F	IPR000468	PF00545	3981	788	19.8 (1)
1XOU	A	IPR005095	PF11439	985	0	PF04888 10.0
	B	IPR021545	PF03433	71	0	PF01464 7.0
1ZHH	A	IPR025997	PF09308	-		
	B	IPR015387	PF13407	687	132	19.2 (1)
1ZNV	A, C	IPR000290	-			
	B, D	IPR003060	PF01320	408	196	48.0 (1)
1ZUN	A	IPR011784	PF00009	5643	1057	18.7 (1)
	B	IPR011779	PF01507	13199	3400	25.8 (1)
2B59, 2CCL	A	IPR002102	PF00404	5322	5	0.1 (>10)
	B	IPR016134	PF00963	8895	67	0.8 (3)
	B	IPR018247	PF00963	-		
	B	IPR002105	PF00963	2044	17	0.8 (4)
2B5U	A, C	IPR024575	PF03513	109	18	16.5 (3)
	B, D	IPR003063	PF11570	227	0	PF06958-PF09000 20.0
	B, D	IPR003063	PF09000	227	45	19.8 (1)
	B, D	IPR003063	PF06958	227	45	19.8 (1)
2C1D	A, C, E, G	IPR025710	PF00034	6664	642	9.6 (3)
	B, D, F, H	IPR030999	-			
2CNW	A, B, C	IPR022941	PF00448	804 (4 nodes)	0	PF00886 33.0
	A, B, C	IPR004780	PF00448	1203	0	PF00886 29.0
	A, B, C	IPR022941	PF02881	804 (4 nodes)	0	PF00886 33.0
	A, B, C	IPR004780	PF02881	1203	0	PF00886 29.0
	D, E, F	IPR004390	PF00448	5252	565	10.8 (2)
	D, E, F	IPR004390	PF02881	5252	565	10.8 (2)
2CO6, 2CO7	A	IPR018569	PF00345	226	21	9.3 (1)
	A	IPR018569	PF02753	226	21	9.3 (1)
	B	IPR001829	-	14379	-	-
2CYZ, 2CZ0, 2CZ1, 62CZ, 2CZ7, 2D0Q	A	IPR023900	PF02211	1508	719	47.7 (1)
	A	IPR018141	PF02211	1339	641	47.9 (1)
	B	IPR003168	PF02979	1390	522	37.6 (1)
2DFX	E	IPR021964	PF11480	19 (1 node)	19	100 (1)
	I	IPR020127	PF12106	9 (1 node)	0	PF00892 100.0
2ERH, 2WPT, 3U43	A	IPR000290	-	763	-	-
	B	IPR003060	PF01320	408	196	48.0 (1)
2ES4	A, B	IPR000073	PF03280	-		

	D, E	IPR004961	PF12697	4353	22	0.5 (>10)
	D, E	IPR004961	PF00561	4353	643	14.8 (1)
	D, E	IPR004961	PF05057	4353	2	0.05 (>10)
	D, E	IPR004961	PF01674	4353	5	0.1 (>10)
2F9Z	A, B	IPR007597	PF03975	29480	911	3.1 (10)
	C, D	IPR005659	PF04509	22051	799	3.6 (7)
2FHZ	B	IPR021964	PF11480	19 (1 node)	19	100 (1)
	A	IPR020127	PF12106	9 (1 node)	0	PF00892 100.0
2FRV	I, S	IPR001821	PF00374	7492	1388	18.5 (1)
	J, L	IPR001501	PF01058	16555	2163	13.1 (1)
	B, D, F, H, J, L	IPR001501	PF14720	16555	1313	7.9 (3)
2G38	A, C	IPR000084	PF00823	8080	277	3.43 (3)
	B, D	IPR000030	PF00934	7962	1087	13.65 (1)
2GRX	A, B	IPR010105	PF16031	-		
	A, B	IPR010105	PF03544	-		
	C, D	IPR003538	PF07715	27549	1040	3.8 (3)
	C, D	IPR003538	PF00593	27549	1040	3.8 (3)
2GSK	A	IPR010101	PF03544	3853	0	PF01177 7.8
	B	IPR003538	PF07715	27549	1040	3.8 (3)
	B	IPR003538	PF00593	27549	1040	3.8 (3)
2GSM, 3DTU, 3FYE, 3FYI	A, C	IPR000883	PF00116-PF02790	-		
	A, C	IPR014241	PF00116-PF02790	16474	638	3.9 (6)
	B, D	IPR002429	PF00115	11968	3276	27.4 (1)
	B, D	IPR011759	PF00115	11787	2092	17.7 (1)
	B, D	IPR014222	PF00115	10264	1647	16.0 (1)
	B, D	IPR001505	PF00115	8677	2246	25.9 (1)
2GYK, 2GZE, 2GZF, 2GZG, 2GZI, 2GZJ	A,C	IPR000290	-			
	B,D	IPR003060	PF01320	408	196	48.0 (1)
2H9A	A	IPR016218	PF03599	1596	274	17.2 (1)
	B	IPR016041	PF03599-PF04060	4672	414	8.9 (5)
2J7P	A, B	IPR022941	PF00448-PF02881	804 (4 nodes)	0	PF00886 33.0
	A, B	IPR004780	PF00448-PF02881	1203 (5 nodes)	0	PF00886 29.0

	D, E	IPR004390	PF00448-PF02881	5252	565	10.1 (2)
2O3B	A	IPR001604	PF07924	5570	90	1.6 (>10)
	A	IPR020821	PF07924	4223	90	3.4 (>10)
	B	IPR012489	PF01223	651	91	14.0 (1)
2OZ1	A, C, E, G	IPR025710	PF00034	6664	642	9.6 (3)
	B, D, F, H	IPR030999	-			
2QDY, 2ZPB, 2ZPE, 2ZPF, 2ZPG, 2ZPH, 2ZPI	A, C, E, G	IPR023900	PF02211	1508	719	47.7 (1)
	A, C, E, G	IPR018141	PF02211	1339	641	47.9 (1)
	B, D, F, H	IPR003168	PF02979	1390	522	37.6 (1)
2UY7	A, C, E, G	IPR001829	PF00419	14379	3544	24.6 (2)
	B, D, F, H	IPR000259	PF00345	35565	5914	16.6 (1)
	B, D, F, H	IPR000259	PF02753	35565	5914	16.6 (1)
2VLN, 2VLO, 2VLP, 2VLQ	A	IPR000290	-	763	-	-
	B	IPR003060	PF01320	408	196	48.0 (1)
2VN5, 4DH2	A, C	IPR002102	PF00404	5322	5	0.09 (>10)
	B, D	IPR016134	PF00963	8895	67	0.8 (3)
	B, D	IPR002105	PF00963	2044	17	0.8 (4)
2VN6	A	IPR002102	PF00404	5322	5	0.09 (>10)
	B	IPR016134	PF00963	8895	67	0.8 (3)
	B	IPR002105	PF00963	2044	17	0.8 (4)
2W2D, 2XHL	A, C	IPR000395	PF07952	111	10	9.0 (8)
	B, D	IPR012500	PF01742	42	11	26.2 (1)
2WJZ, 3ZR4	A, C, E	IPR006062	PF00117	6166	984	16.0 (1)
	A, C, E	IPR004651	PF00117	645	281	43.6 (1)
	B, D, F	IPR010139	PF00977	2277	856	37.6 (1)
2WPN, 3ZE7	A	IPR001821	PF00374	7492	1388	18.5 (1)
	B	IPR001501	PF01058	16555	2163	13.1 (1)
	B	IPR001501	PF14720	16555	1313	7.9 (3)
2Y3N	A, C	IPR002102	-			
	B, D	IPR016134	PF00963	8895	67	0.8 (3)
	B, D	IPR018247	PF00963	-		
2YCL	A	IPR016218	PF03599	1596	274	17.2 (1)
	B	IPR016041	PF04060	4672	414	8.9 (5)
	B	IPR016041	PF03599	4672	414	8.9 (5)
2ZMX, 2ZMY, 2ZMZ	A	IPR002227	PF06236	6365	136	2.1 (3)

	B	IPR010928	PF00264	2750	334	12.1 (1)
2ZWD, 2ZWE, 2ZWF, 2ZWG 2AHK, 2AHL 2ZMX, 2ZMY, 2ZMZ	A	IPR002227	PF06236	6365	136	2.1 (3)
	B	IPR010928	PF00264	2750	334	12.1 (1)
3A8L, 3A8M, 3A8O, 3WVD, 3WVE, 3X28	A,C,E,G	IPR023900	PF02211	1508	719	47.7 (1)
	A	IPR018141	PF02211	1339	641	47.9 (1)
	B,D, F, H	IPR003168	PF02979	1390	522	37.6 (1)
3AON	A	IPR002699	PF01990	10446	1102	10.5 (5)
	B	IPR008218	PF01813	9527	836	8.8 (5)
	B	IPR022944	PF01813	2733	313	11.5 (5)
3AWS, 3AWT, 3AWU, 3AWV, 3AWW, 3AWX, 3AWY, 3AWZ, 3AX0	A	IPR002227	PF06236	6365	136	2.1 (3)
	B	IPR010928	PF00264	2750	334	12.1 (1)
3BTP	A	IPR009868	PF12189	92	7	7.6 (6)
	B	IPR024237	PF07229	53	5	9.4 (1)
3CLR, 3CLS, 3CLT, 3CLU	C	IPR012255	PF00766	3430	1273	37.1 (1)
	C	IPR012255	PF01012	3430	1273	37.1 (1)
	D	IPR001308	PF01012	3625	1223	33.7 (1)
3CUR	A, B, C	IPR001821	PF00374	7492	1388	18.5 (1)
	H, I, J	IPR001501	PF01058	16555	2163	13.1 (1)
	H, I, J	IPR001501	PF14720	16555	1313	7.9 (3)
3CUS, 3H3X	A, B, C	IPR001821	PF00374	7492	1388	18.5 (1)
	Q, R, S	IPR001501	PF01058	16555	2163	13.1 (1)
	Q, R, S	IPR001501	PF14720	16555	1313	7.9 (3)
3DA7	A, B, E, G	IPR000026	PF01337	2114	1184	56.0 (1)
	A, B, E, G	IPR001887	PF01337	1854	419	22.6 (1)
	C, D, F, H	IPR000468	-			
3DBO	A	IPR006442	PF01850	30684	4282	14.0 (1)
	B	IPR022907	PF02604	-		
3DDR	A, B	IPR011276	-			
	A, B	IPR010949	-			
	C, D	IPR010495	PF07715	1731	118	6.8 (1)
	C, D	IPR010495	PF00593	1731	118	6.8 (1)
	C, D	IPR010495	PF07660	1731	10	0.6 (>10)
3E2K, 3GMW, 3N4I	A, B	IPR000871	-			
	C, D	IPR009099	PF13354	90	0	PF00005 12.0
3E9J	B, E	IPR023205	PF02600	9016	70	0.8 (>10)

	C, F	IPR022920	PF01323	4316	9	0.2 (>10)
	C, F	IPR003752	PF01323	16236	60	0.4 (>10)
3EGV	A	IPR004498	PF00298	3416	0	PF04452 15
	A	IPR004498	PF03946	3416	0	PF04452 15
	B	IPR000911	PF13649	18975	4	0.02 (>10)
	B	IPR000911	PF06325	18975	0	PF00687 12.0
	B	IPR000911	PF01555	18975	3	0.02 (>10)
	B	IPR000911	PF05175	18975	18	0.1 (>10)
	B	IPR000911	PF08241	18975	5	0.03 (>10)
	B	IPR000911	PF13847	18975	3	0.02 (>10)
	B	IPR006519	PF13649	17926	2	0.01 (>10)
	B	IPR006519	PF06325	17926	0	PF00687 13.0
	B	IPR006519	PF01555	17926	0	PF00687 13.0
	B	IPR006519	PF05175	17926	15	0.08 (>10)
	B	IPR006519	PF08241	17926	1	0.006 (>10)
	B	IPR006519	PF13847	17926	0	PF00687 13.0
3EJB, 3EJD, 3EJE	A, C, E, G	IPR003231	PF00067	5965	20	0.3 (>10)
	B, D, F, H	IPR001128	PF00550	-		
	B, D, F, H	IPR002397	PF00550	-		
3FAV	A, C	IPR010310	PF06013	26102	2241	8.6 (1)
	B, D	IPR010310	PF06013	26102	2241	8.6 (1)
3GFK, 3IHQ	A	IPR006504	-			
	A	IPR023731	-			
	A	IPR006660	-			
	B	IPR011773	PF03960	7202	0	PF01196 11.0
3GKL	A, B	IPR003060	PF01320	408	196	48.0 (1)
	C, D	IPR000290	-			
3GQB	A, C	IPR022878	PF00006	6675	1118	16.7 (2)
	A, C	IPR022878	PF02874	6675	1118	16.7 (2)
	B, D	IPR022879	PF16886	9067	1118	12.3 (2)
	B, D	IPR022879	PF00006	9067	1348	14.9 (2)
	B, D	IPR022879	PF02874	9067 (9 nodes)	1348	14.9 (2)
3GTY	X	IPR005215	-			
	S	IPR005717	PF05698	22154	5	0.02 (>10)
	S	IPR005717	PF05697	22154	5	0.02 (>10)
	S	IPR000235	PF05698	26333	5	0.02 (>10)

	S	IPR000235	PF05697	26333	5	0.02 (>10)
3GYX	A, C, E, G, I, K	IPR011803	PF13187	607	216	35.6 (1)
	A, C, E, G, I, K	IPR011803	PF12139	607	216	35.6 (1)
	B, D, F, H, J, L	IPR011802	PF00890	611	168	27.5 (1)
3HI2	A, C	IPR032758	PF15723	1372	336	24.5 (1)
	B, D	IPR031451	-			
3HZH	A	IPR001789	PF04509			
	B	IPR038756	PF00072	3669	510	13.9 (1)
3K1I	A, B	IPR003713	PF16522	24549	0	PF00669-PF00700 9.0
	C, D	IPR032411	PF02561	530	1	0.2 (>10)
3KCP	A	IPR016134	PF00963	8895	67	0.8 (3)
	A	IPR002105	PF00963	2044	17	0.8 (4)
	A	IPR018247	PF00963	-		
	A	IPR002102	PF00963	5322	245	4.6 (1)
	B	IPR002102	PF00963	5322	245	4.6 (1)
3KOW, 3KOY, 3KOZ, 3KP0	A, B, C, D	IPR015130	PF16552	2296	149	6.5 (7)
	A, B, C, D	IPR028991	PF16552	1398	145	10.4 (4)
	A, B, C, D	IPR006158	PF16552	-		
	E, F, G, H	IPR015130	PF02310	2296	295	12.8 (1)
	E, F, G, H	IPR015130	PF16554	2296	295	12.8 (1)
	E, F, G, H	IPR015130	PF09043	2296	138	6.0 (8)
3LB8	A, B	IPR028202	PF00111	1867	3	0.2 (>10)
	A, B	IPR023753	PF00111	-		
	C, D	IPR001055	PF00070	3269	0	PF00266 21.0
	C, D	IPR001055	PF07992	3269	596	18.2 (3)
	C, D	IPR001055	PF14759	3269	596	18.2 (3)
3LCB	A, B	IPR010452	PF00180	704	0	PF00108-PF02803 34.0
	C, D	IPR004439	PF06315	76	0	PF06183 6.6
3ME0	A	IPR001829	PF03628	14379	0	PF00577-PF13953-PF13954 25.0
	B	IPR005309	PF00345	211	20	9.5 (2)
	B	IPR005309	PF02753	211	20	9.5 (2)
3ML1, 3O5A	A	IPR010051	-			
	B	IPR005591	PF00384	4036	550	13.6 (2)
	B	IPR005591	PF04879	4036	550	13.6 (2)
	B	IPR005591	PF01568	4036	550	13.6 (2)

3MYR	A, C, E, G	IPR001821	PF00374	7492	1388	18.5 (1)
	B, D, F, H	IPR001501	PF01058	16555	2163	13.1 (1)
	B, D, F, H	IPR001501	PF14720	16555	1313	7.9 (3)
3NQY, 3NQZ	A	IPR011096	PF02868	3591	20	0.6 (>10)
	A	IPR011096	PF01447	3591	20	0.6 (>10)
	A	IPR025711	PF02868	37917	1	0.003 (>10)
	A	IPR025711	PF01447	37917	1	0.003 (>10)
	B	IPR023612	PF03413	4593	20	0.4 (>10)
	B	IPR023612	PF07504	4593	20	0.4 (>10)
3NY7	A	IPR002645	PF00550	-		
	B	IPR003231	PF01740	5965	3	0.05 (>10)
3O3M, 3O3N, 3O3O	A, C	IPR010327	PF06050	8834	1861	21.1 (1)
	B, D	IPR010327	PF06050	8834	1861	21.1 (1)
3OCD	A, C	IPR025710	PF00034	6664	642	9.6 (3)
	B, D	IPR030999	-			
	B, D	IPR016823	-			
3OGI, 4GZR	A, C	IPR010310	PF06013	26102	2241	8.6 (1)
	A, C	IPR009416	PF06013	12	2	16.7 (1)
	B, D	IPR010310	PF06013	26102	2241	8.6 (1)
3OM3, 3OMA, 3OMI, 3OMN	A, C	IPR000883	PF00116-PF02790	-		
	B, D	IPR001505	PF00115	8677	2246	25.9 (1)
	B, D	IPR002429	PF00115	11968	3276	27.4 (1)
	B, D	IPR011759	PF00115	11787	2092	17.7 (1)
	B, D	IPR014222	PF00115	10264	1647	16.0 (1)
3OUR	A, C, E, G	IPR010520	-			
	B, D, F, H	IPR001127	PF06500	4740	0	PF00232 18.0
	B, D, F, H	IPR001127	PF00561	4740	5	0.1 (>10)
3PH0	A, B	IPR012671	-			
	C, D	IPR013348	PF08988	364	22	6.0 (>10)
3PNT	A, C	IPR010900	PF16718	41 (4 nodes)	21	51.2 (1)
	B, D	IPR032002	-			
3PRO	A, B	IPR001316	PF02983	4588	189	4.1 (5)
	C, D	IPR004236	PF00089	4542	104	2.3 (8)
3QHY	A	IPR000871	PF13540	4478	0	PF00126-PF03466 15.0
	B	IPR000408	PF13354			

3REQ, 4REQ	A	IPR006099	PF01642	2533	878	34.7 (1)
3REQ, 4REQ	A	IPR006098	PF01642	842	42	5.0 (4)
3REQ, 4REQ	A	IPR006159	PF01642	4982	1627	32.7 (1)
3REQ, 4REQ	A	IPR006158	PF01642	-		
3REQ, 4REQ	B	IPR004608	PF01642	952	294	30.9 (1)
3REQ, 4REQ	B	IPR004608	PF02310	952	294	30.9 (1)
3RGW, 4TTT, 4IUB, 4IUC, 4IUD, 4U9H, 4U9I	S	IPR001821	PF00374	7492	1388	18.5 (1)
3RGW, 4TTT, 4IUB, 4IUC, 4IUD, 4U9H, 4U9I	L	IPR001501	PF01058	16555	2163	13.1 (1)
3RGW, 4TTT, 4IUB, 4IUC, 4IUD, 4U9H, 4U9I	L	IPR001501	PF14720	16555	1313	7.9 (3)
3SQB	A, C, E, G	IPR001829	-			
3SQB	B, D, F, H	IPR000259	PF00345	35565	5914	16.6 (1)
3SQB	B, D, F, H	IPR000259	PF02753	35565	5914	16.6 (1)
3TGO	A, B	IPR017689	PF06804	31760	3	0.009 (>10)
3TGO	C, D	IPR014524	PF13525	2520	0	PF00701 8.3
3TGO	C, D	IPR010653	PF13525	12004	29	0.2 (>10)
3THO	A	IPR027050	PF00149			
3THO	B	IPR004593	PF13558	4651	2751	59.1 (1)
3UL4, 4UYQ	A	IPR002102	PF00404	5322	5	0.09 (>10)
3UL4, 4UYQ	B	IPR016134	PF00963	8895	67	0.8 (3)
3UL4, 4UYQ	B	IPR002105	PF00963	2044	17	0.8 (4)
3UQY, 3USC, 3USE	S, T	IPR001821	PF00374	7492	1388	18.5 (1)
3UQY, 3USC, 3USE	L, M	IPR001501	PF01058	16555	2163	13.1 (1)
3UQY, 3USC, 3USE	L, M	IPR001501	PF14720	16555	1313	7.9 (3)
3UZ0	A, C	IPR024232	PF01551	12988	1	0.008 (>10)
3UZ0	B, D	IPR016047	-			
3V6I	A, Y	IPR002842	PF16999	11245	85	0.8 (10)
3V6I	B, B	-	PF01991			
3VEP	A, D, E, H	IPR039425	PF16751	-		
3VEP	C, G, J, X	IPR031928	PF08281	3784	329	8.7 (5)
3VRD	A	IPR024167	PF07992	11026	96	0.9 (<10)
3VRD	A	IPR024167	PF09242	11026	96	0.9 (<10)
3VRD	B	IPR015323	-			
3VRD	B	IPR023753	-			
3W9C	A	IPR001128	PF00111	-		
3W9C	A	IPR002397	PF00111	-		
3W9C	B	IPR001055	PF00067	3269	669	20.5 (2)

3WDG	A	IPR002043	PF06106	1673	0	PF00005 2.5
	B	IPR009295	PF03167	87	0	PF06124 21.0
3WXX	A, C, E, G	IPR016379	PF04888	2352	151	6.4 (1)
	A, C, E, G	IPR005415	PF04888	2276	236	10.4 (1)
	B, D, F, H	IPR006972	-			
3ZDF	A, B	IPR006424	-			
	A, B	IPR020831	-			
	C, D	IPR003823	PF02800	491	0	PF11375 12.0
	C, D	IPR003823	PF00044	491	0	PF11375 12.0
3ZET, 3ZEU	A	IPR022496	PF00814	11086	940	8.5 (3)
	B	IPR022450	PF00814	661	1	0.2 (>10)
	B	IPR017861	PF00814	2569	4	0.2 (>10)
4AWX	A	IPR006073	PF09012	-		
	A	IPR030389	PF09012	3555	0	PF04023 56.0
	B	IPR023732	-			
4AYF, 4AZ8	A	IPR001829	PF09255	14379	0	PF00577-PF13953-PF13954 25.0
	B	IPR015335	PF00345	26	1	3.8 (2)
	B	IPR015335	PF02753	26	1	3.8 (2)
4BMP	A	IPR000358	PF07972	2022	5	0.2 (>10)
	A	IPR026494	PF07972	2736	506	18.5 (2)
	A	IPR033909	PF07972	627	1	0.2 (>10)
	B	IPR004465	PF00268	6440	1011	15.7 (2)
	B	IPR020852	PF00268	3774	781	20.7 (2)
4C3O	B, D, F	IPR001821	PF00374	7492	1388	18.5 (1)
	A, C, E	IPR001501	PF01058	16555	2163	13.1 (1)
	A, C, E	IPR001501	PF14720	16555	1313	7.9 (3)
4CTA	A, B	IPR008135	PF00994	10073	0	PF00154 12.0
	A, B	IPR008135	PF02464	10073	0	PF00154 12.0
4CXF	A	IPR000838	PF17524	2820	0	PF00005 2.6
	A	IPR013249	PF17524	-		
	A	IPR014284	PF17524	-		
	A	IPR007627	PF17524	-		
	B	IPR035230	PF08281	75	5	6.7 (4)
	B	IPR035230	PF04542	75	5	6.7 (4)
4DH2	A, C	IPR002102	PF00404	5322	5	0.09 (>10)

	B, D	IPR016134	PF00963	8895	67	0.8 (3)
	B, D	IPR002105	PF00963	2044	17	0.8 (4)
4DRF, 4E6N	A, C	IPR032390	-			
	A, C	IPR032380	-			
	B, D	IPR026610	PF16542	1409	646	45.8 (1)
	B, D	IPR026610	PF16536	1409	646	45.8 (1)
	B, D	IPR024026	PF16542	1182 (4 nodes)	646	54.7 (1)
	B, D	IPR024026	PF16536	1182 (4 nodes)	646	54.7 (1)
4DWH	A,C,E,G	IPR001829	-			
	B,D,F,H	IPR000259	PF00345	35565	5914	16.6 (1)
	B,D,F,H	IPR000259	PF02753	35565	5914	16.6 (1)
4EMJ	A	IPR028202	PF00355	1867	403	21.6 (1)
	A	IPR023753	PF00355	-		
	B	IPR017941	PF07992	-		
	B	IPR017941	PF14759	-		
4ETW	A, C	IPR010076	PF00550	6706	0	PF00156 6.5
	B, D	IPR003231	PF00561	5965	0	PF00109-PF02801 8.9
4F48	A	IPR001633	PF07238	-		
	B	IPR009875	PF00563	103403	104	0.1 (>10)
4FOU	A, B	IPR001633	PF07238	-		
	C, D	IPR009875	PF00563	103403	104	0.1 (>10)
4FQ0	A, B	IPR001689	PF14841	30972	817	2.6 (>10)
	C, D	IPR000090	PF02154	27584	848	3.1 (>10)
4G7X	A	IPR032014	PF06519	41 (8 nodes)	0	PF07459 17.0
	B	IPR014161	-			
4GF3	A	IPR010261	-			
	B	IPR015103	PF05932	102	5	4.9 (1)
4HFF	A	IPR025562	PF16695	1135	90	7.9 (1)
	B	IPR032032	PF14113	2210	133	6.0 (1)
4HG6	A	IPR003919	PF03170	4226	788	18.6 (2)
	A	IPR005150	PF03170	2802	530	18.9 (2)
	B	IPR018513	PF07238	10336	679	6.6 (3)
	B	IPR018513	PF00535	10336	679	6.6 (3)
4HLU	A, B	IPR017871	PF00005	-		
	A, B	IPR003593	PF00005	-		
	A, B	IPR003439	PF00005	-		

	C, D	IPR030947	PF00005	16177	1453	9.0 (2)
4HN4, 4HPJ, 4HPX, 4HT3, 4KKX	A	IPR002028	PF00291	5033	1056	21.0 (1)
	B	IPR006654	PF00290	602	287	47.7 (1)
	B	IPR023026	PF00290	468	184	39.3 (1)
4I0X	A, C, E, G, I, K	IPR010310	PF06013	26102	2241	8.6 (1)
	B, D, F, H, J, L	IPR010310	PF06013	26102	2241	8.6 (1)
4IEF	A, C, E, G	IPR012600	PF01364	126	0	PF13860 3.2
	A, C, E, G	IPR012600	PF03785	126	0	PF13860 3.2
	B, D, F, H	IPR005536	PF08126	226	1	0.4 (>10)
	B, D, F, H	IPR001769	PF08126	2283	0	PF03781 22.2
	B, D, F, H	IPR039392	PF08126	66	5	7.6 (1)
4IGL	A, C	IPR003284	PF18807	135	0	PF00005 3.0
	B, D	IPR022385	PF12256	-		
	B, D	IPR022385	PF03534	-		
	B, D	IPR022385	PF12255	-		
4IW3	A, J	IPR006620	PF03144	4855	1	0.02 (>10)
	A, J	IPR006620	PF00009	4855	2	0.04 (>10)
	A, J	IPR006620	PF03143	4855	0	PF07690 5.2
	A, J	IPR005123	PF03144	-		
	A, J	IPR005123	PF00009	-		
	A, J	IPR005123	PF03143	-		
	B, K	IPR004541	PF13640	13404	0	PF00164 6.1
4JWS, 4JWU, 4JX1	A, B	IPR001128	PF00111	-		
	A, B	IPR002397	PF00111	-		
	C, D	IPR001055	PF00067	3269	669	20.5 (2)
4KBM	A	IPR015712	PF02559	-		
	B	IPR003711	PF04561	1762	0	PF01195 35.0
4KL8, 4KN9, 4KO1, 4KO2, 4KO3, 4KO4	S, T	IPR001821	PF00374	7492	1388	18.5 (1)
	L, M	IPR001501	PF01058	16555	2163	13.1 (1)
	L, M	IPR001501	PF14720	16555	1313	7.9 (3)
4KPU, 4L2I	A,C	IPR001308	PF01012	3625	1223	33.7 (1)
	B,D	IPR012255	PF00766	3430	1273	37.1 (1)
	B,D	IPR012255	PF01012	3430	1273	37.1 (1)
4KT6	A,C	IPR010900	PF16718	41 (4 nodes)	21	51.2 (1)
	B,D	IPR032002	-			
4LO8	A, C, E, G	IPR003897	-			

	B, D, F, H	IPR008903	PF17993	18	0	PF01742-PF07953-PF08470 17.0
4MRT	A	IPR004568	PF00550	3488	80	2.3 (4)
	A	IPR008278	PF00550	18829	437	2.3 (1)
	C	IPR009081	PF01648	-		
	C	IPR020806	PF01648	-		
4N4R	A, C	IPR020889	PF04390	20993	1	0.005 (>10)
	B, D	IPR007485	PF04453	43556	21	0.05 (>10)
	B, D	IPR007485	PF03968	43556	21	0.05 (>10)
4NRH	A, C	IPR013401	PF07720	3232	43	1.3 (>10)
	A, C	IPR010812	PF07720	4718	56	1.2 (>10)
	B, D	IPR005415	-			
4O93, 4O9P	A, C	IPR024605	-			
	B, D	IPR034300	PF12769	5068	780	15.4 (3)
4O9T	E, G	IPR024605	-			
	F, H	IPR034300	PF12769	5068	780	15.4 (3)
4OB0, 4OB2, 4OB3	A,C,E,G	IPR023900	PF02211	1508	719	47.7 (1)
	A	IPR018141	PF02211	1339	641	47.9 (1)
	B,D, F, H	IPR003168	PF02979	1390	522	37.6 (1)
4OB1	A	IPR018141	PF02211	1339	641	47.9 (1)
	B	IPR003168	PF02979	1390	522	37.6 (1)
4OUD	A	IPR024107	PF00579	13308	22	0.2 (>10)
	A	IPR024088	PF00579	174	0	PF00905-PF00912 92.0
	A	IPR002305	PF00579	-		
	A	IPR002307	PF00579	342	0	PF00905-PF00912 46.0
	B	IPR024107	PF01479	13308	213	1.6 (>10)
	B	IPR024107	PF00579	13308	22	0.2 (>10)
	B	IPR024088	PF01479	174	0	PF00905-PF00912 92.0
	B	IPR024088	PF00579	174		PF00905-PF00912 92.0
	B	IPR002305	PF01479	-		
	B	IPR002305	PF00579	-		
	B	IPR002307	PF01479	342	0	PF00905-PF00912 46.0
	B	IPR002307	PF00579	342	0	PF00905-PF00912 46.0
4P3Y	A	IPR004541	PF01323	13404	0	PF00164 6.0
	B	IPR023205	PF03144	9016	5	0.06 (>10)
	B	IPR023205	PF00009	9016	6	0.07 (>10)

	B	IPR023205	PF03143	9016	1	0.01 (>10)
4PC1, 4PC2, 4PC3, 4PC6	A, B	IPR004541	PF00889	13404	0	PF00164 6.0
	C, D	IPR001816	PF03144	2334	0	PF00318 32.0
	C, D	IPR001816	PF00009	2334	1	0.04 (>10)
	C, D	IPR001816	PF03143	2334	1	0.04 (>10)
4PC7	A	IPR004541	PF00889	13404	0	PF00164 6.0
	C	IPR001816	PF03144	2334	0	PF00318 32.0
	C	IPR001816	PF00009	2334	1	0.04 (>10)
	C	IPR001816	PF03143	2334	1	0.04 (>10)
4PW9	A	IPR008335	PF13442	2353	158	6.7 (2)
	B	IPR009056	PF03404	-		
	B	IPR009056	PF00174	-		
4Q35	A	IPR020889	PF04390	20993	1	0.005 (>10)
	B	IPR007485	PF03968	43556	21	0.05 (>10)
	B	IPR007485	PF04453	43556	21	0.05 (>10)
4QLP	A	IPR028953	PF14021	171	46	26.9 (1)
	B	IPR025331	PF15598	2926	5	0.2 (>10)
4R40	A, C	IPR014167	-			
	B, D	IPR039001	PF04052	17121	2250	13.1 (1)
	B, D	IPR039001	PF07676	17121	2250	13.1 (1)
4RHB	A,C	IPR020889	PF04390	20993	1	0.005 (>10)
	B,D	IPR007485	PF04453	43556	21	0.05 (>10)
4TKV	A,C	IPR010143	PF00148	2184 (7 nodes)	514	23.5 (2)
	A,C	IPR010143	PF11844	2184 (7 nodes)	207	9.5 (5)
	A,C	IPR005972	PF00148	2689	324	12.0 (2)
	A,C	IPR005972	PF11844	2689	188	7.0 (3)
	B,D	IPR005976	PF00148	2689	324	12.0 (2)
4TPS	A,C	IPR019683	PF11638	2326	0	PF03672 7.9
	B,D	IPR001957	PF10747	11780	0	PF00712-PF02767-PF02768 12.0
4TXV	A, C	IPR013766	PF00116	-		
	A, C	IPR013740	PF00116	7666	4	0.05 (>10)
	B, D	IPR002429	PF08534	11968	6	0.05 (>10)
	B, D	IPR034210	PF08534	8061	0	PF00510 10.0
4U39	A, B, C, D, E, F, G, H, I	IPR000158	PF13072	5786	0	PF02491-PF14450 14.6

	J, K, L, M, N, O, P, Q, R	IPR025177	PF00091	1674	0	PF01944 6.7
4U3S	A	IPR002102	PF13620	5322	5	0.09 (>10)
	B	IPR016134	PF00963	8895	67	0.8 (3)
	B	IPR018247	PF00963	-		
4UD2, 4UD6	A, B, C	IPR001821	PF00374	7492	1388	18.5 (1)
	Q, R, S	IPR001501	PF01058	16555	2163	13.1 (1)
	Q, R, S	IPR001501	PF14720	16555	1313	7.9 (3)
4UDM	A	IPR003063	PF09000	227	45	19.8 (1)
	B	IPR009105	PF03513	574	74	12.9 (1)
4UE2, 4UE6, 4UEQ, 4UEW, 4UCQ, 4UCW, 4UCX, 4UPE, 4URH	A,B,C	IPR001821	PF00374	7492	1388	18.5 (1)
	Q,R,S	IPR001501	PF01058	16555	2163	13.1 (1)
	Q,R,S	IPR001501	PF14720	16555	1313	7.9 (3)
4UHP	E, G	IPR000290	-			
	F, H	IPR003060	PF01320	408	196	48.0 (1)
4UPV, 4UQL, 4UQP	A, B	IPR001821	PF00374	7492	1388	18.5 (1)
	Q, R	IPR001501	PF01058	16555	2163	13.1 (1)
	Q, R	IPR001501	PF14720	16555	1313	7.9 (3)
4UQZ	A	IPR009211	PF05591	5731	472	8.2 (6)
	B	IPR008312	PF07024	12972	241	1.9 (>10)
	B	IPR008312	PF14559	12972	0	PF05943 10.0
4UTQ	A	IPR019998	PF00137	7972	0	PF00825 17.0
	A	IPR001708	PF00137	-		
	Z	IPR000454	PF14849	22104	3	0.01 (>10)
	Z	IPR000454	PF02096	22104	3	0.01 (>10)
	Z	IPR005953	PF14849	20151	5	0.02 (>10)
	Z	IPR005953	PF02096	20151	5	0.02 (>10)
4W4K	A, C	IPR000084	PF00823	8080	277	3.4 (3)
	B, D	IPR000030	-			
4WI0	A	IPR002102	PF13620	5322	5	0.09 (>10)
	B	IPR016134	PF00963	8895	67	0.8 (3)
	B	IPR018247	PF00963	-		
	B	IPR002105	PF00963	2044	17	0.8 (4)
4WQ4, 4WQ5, 4YDU	A, B	IPR022450	PF00814	661	1	0.2 (>10)
	A, B	IPR017861	PF00814	2569	4	0.2 (>10)
	C, D	IPR022496	PF00814	11086	940	8.5 (3)

4XGA	A	IPR017687	PF07244	13524	0	PF09976 11.0
	B	IPR039910	PF01011	-		
	B	IPR023707	PF01011	18523	0	PF03938 13.0
4XWJ	A	IPR007448	PF00381	2301	0	PF01208 8.3
	A	IPR023785	PF00381	807	0	PF00216. PF02581. PF01208 6.0
	B	IPR000032	PF04353	3534	0	PF00391-PF02896-PF05524 13.0
	B	IPR001020	PF04353	1719	0	PF00391-PF02896-PF05524 18.0
	B	IPR002114	PF04353	4892	0	PF00391-PF02896-PF05524 20.0
4Y7O	A, B	IPR010623	-			
	C, D	IPR017734	PF06744	18036	1410	7.8 (7)
4YLF	A, C	IPR012165	PF13450	6157	1	0.02 (>10)
	A, C	IPR012165	PF13738	6157	1	0.02 (>10)
	A, C	IPR012165	PF07992	6157	3	0.05 (>10)
	A, C	IPR012165	PF00070	6157	0	PF01180 16.0
	A, C	IPR012165	PF14691	6157	0	PF01180 16.0
	B, D	IPR006004	PF00970	2026	10	0.5 (>10)
	B, D	IPR006004	PF00175	2026	580	28.6 (1)
	B, D	IPR006004	PF10418	2026	580	28.6 (1)
4YRY	A, C	IPR012165	PF13738	6157	1	0.02 (>10)
	A, C	IPR012165	PF07992	6157	3	0.05 (>10)
	A, C	IPR012165	PF14691	6157	0	PF01180 16.0
	B, D	IPR006004	PF00970	2026	10	0.5 (>10)
	B, D	IPR006004	PF00175	2026	580	28.6 (1)
	B, D	IPR006004	PF10418	2026	580	28.6 (1)
4ZGD, 4ZGE	A,C,E,G,I,K,M,O	IPR004232	PF02211	1121	883	78.8 (1)
	B,D,F,H,J,L,N,P	IPR003168	PF02979	1390	522	37.6 (1)
4ZGJ, 4FM4	A,C,E,G,I,K,M,O	IPR023900	PF02211	1508	719	47.7 (1)
	B,D,F,H,J,	IPR003168	PF02979	1390	522	37.6 (1)
5CEC, 5CED, 5CER	A	IPR000667	PF12796	8149	11	0.13 (>10)
	B	IPR020683	PF02113			
	B	IPR002110	PF02113			

Additional Table 2 Analysis of the ± 3 neighborhood of misclassified interaction partners. The table lists the outcome for those cases, where the known interaction partner reached rank two in an analysis of the ± 10 neighborhood; compare Additional Table 1. For each PDB-ID of the dataset, all chains were identified and the corresponding InterPro families determined. Additionally, Pfam numbers were deduced for the interaction partner. The column "Sum of Pfam occurrences" gives the sum of the absolute frequencies (*SeqCount* values) of all *pf_nodes* from the GNN generated for the InterPro family listed in column three. The column "Occurrence of Interaction Partner" lists the absolute frequency (*SeqCount* value) of the Pfam node representing the known interaction partner (listed in column four) and the last column lists the normalized frequency of this *pf_node* and its rank. If the known interaction partner was not found in the ±3 neighborhood, the Pfam-ID and the relative frequency of this FP is given. If the GNN contained less than 10 nodes, the number of nodes is additionally listed in the column "Sum of Pfam occurrences". Labelled in yellow are the cases, where the rank did not change; a green label indicates that the rank changed to 1 and a red label indicates that the rank was larger than two.

PDB-ID	Chains	InterPro Family of Chains	Pfam of Interaction Partner	Sum of Pfam occurrences	Occurrence of Interaction Partner	Frequency of Interaction Partner [%] (Rank)
2CNW	D, E, F	IPR004390	PF00448	5252	565	10.8 (2)
	D, E, F	IPR004390	PF02881	5252	565	10.8 (2)
2J7P	D, E	IPR004390	PF00448-PF02881	1682	24	1.4 (7)
2UY7	A, C, E, G	IPR001829	PF00419	8881	3109	35.0 (2)
3GQB	A. C	IPR022878	PF00006	6008	1272	21.2 (1)
	A. C	IPR022878	PF02874	6008	1272	21.2 (1)
	B. D	IPR022879	PF16886	3583 (4 nodes)	1044	29.1 (2)
	B. D	IPR022879	PF00006	3583 (4 nodes)	1044	29.1 (2)
	B. D	IPR022879	PF02874	3583 (4 nodes)	1044	29.1 (2)
3ME0	B	IPR005309	PF00345	68	3	4.4 (4)
	B	IPR005309	PF02753	68	3	4.4 (4)
3ML1, 3O5A	B	IPR005591	PF00384	2563	538	21.0 (1)
	B	IPR005591	PF04879	2563	538	21.0 (1)
	B	IPR005591	PF01568	2563	538	21.0 (1)
3W9C	B	IPR001055	PF00067	563	13	2.3 (8)
4AYF, 4AZ8	B	IPR015335	PF00345	3	0	PF00577- PF13953- PF13954 33.0
	B	IPR015335	PF02753	3	0	PF00577- PF13953- PF13954 33.0
4BMP	B	IPR004465	PF00268	6440	1011	15.7 (2)
	B	IPR020852	PF00268	2321	707	30.5 (2)
4HG6	A	IPR003919	PF03170	4226	788	18.6 (2)
	A	IPR005150	PF03170	1579	457	28.9 (1)
4HLU	C, D	IPR030947	PF00005	6807	1407	20.7 (2)
4JWS, 4JWU, 4JX1	C, D	IPR001055	PF00067	563	13	2.3 (8)
4PW9	A	IPR008335	PF13442	815	157	19.3 (1)
4TKV	A, C	IPR010143	PF00148	1471 (4 nodes)	464	31.5 (2)
	A, C	IPR005972	PF00148	2689	324	12.0 (2)
	B, D	IPR005976	PF00148	860	344	40.0 (1)
